# Types of Vascular Access and Associated Clinical Outcomes in Dialysis Patients

**DOI:** 10.7759/cureus.83691

**Published:** 2025-05-07

**Authors:** Diana D Nenova, Yanko G Yankov

**Affiliations:** 1 Second Department of Internal Disease, Medical University "Prof. Dr. Paraskev Stoyanov", Varna, BGR; 2 Clinic of Nephrology and Dialysis, University Hospital "St. Marina", Varna, BGR; 3 Clinic of Maxillofacial Surgery, University Hospital "St. Marina", Varna, BGR; 4 Department of General and Operative Surgery, Medical University "Prof. Dr. Paraskev Stoyanov", Varna, BGR

**Keywords:** adequacy, anemia, arterio-venousus fistula, blood flow, dialisys, mortality, outcome, permanent catheter, quality of life, vasscular access

## Abstract

Background

Vascular access in patients with end-stage renal disease (ESRD) plays a crucial role in determining both the quality of life and survival rates, as it directly affects dialysis effectiveness. It connects the patient to the dialysis machine and significantly impacts the dialysis dose by influencing blood flow efficiency. Despite advancements in technology, improved care, and collaborative efforts by healthcare providers and patients, complications related to vascular access continue to be a major cause of hospitalizations and mortality among patients with ESRD. Our study aims to evaluate how vascular access impacts the dialysis dose and clinical outcomes, with the ultimate target of enhancing patients' quality of life while minimizing costs to the healthcare system. We seek to identify new strategies to reduce complications associated with vascular access, which contribute to inadequate dialysis and higher mortality rates, and to encourage the integration of these strategies into routine clinical practice.

Methodology

This retrospective study was conducted at the Clinic of Nephrology and Dialysis at the University Hospital "St. Marina" in Varna, Bulgaria, over five years, from January 2017 to December 2021. During this period, the medical records and routine laboratory tests of 87 patients who met the study criteria were reviewed. Patients were categorized into two groups based on the type of permanent vascular access: Group 1 included 45 patients with an arteriovenous fistula (AVF), and Group 2 included 42 patients with a permanent tunneled vascular catheter (PC). During the study period, the specified indicators, along with recorded mortality and hospitalization rates, were analyzed in relation to the type of vascular access.

Results

Our analysis demonstrated the significant superiority of AVF in several key areas. Specifically, patients with AVF showed significantly higher dialysis adequacy, as measured by the single-pool Kt/V index (spKt/V) and urea reduction ratio (URR), along with higher serum hemoglobin levels and lower erythropoietin requirements (*P *< 0.0001). The results of our study showed that using PC as vascular access is associated with a significantly higher risk of death, four times greater than in patients with AVF (*P *< 0.0001).

The significantly higher incidence of complications in the PC is well-researched and is directly linked to an increased rate of hospitalizations and mortality in this group. These outcomes are primarily due to access-related events, but also reflect the broader impact of compromised dialysis adequacy. Inadequate dialysis, in turn, worsens clinical outcomes, potentiating issues such as malnutrition, chronic inflammation, and immune dysfunction. These factors collectively contribute to the poor prognosis observed in patients with PC, reinforcing the importance of optimal vascular access in improving patient survival and quality of life.

Conclusions

In conclusion, although indwelling tunneled catheters may be necessary in certain situations, our findings confirm the superior outcomes associated with the use of AVF in terms of dialysis adequacy, survival, anemia control, and overall quality of life, data supported by two large multicenter trials on this topic. AVF placement must be prioritized whenever possible to reduce complications and improve the long-term prognosis of patients with ESRD.

## Introduction

Vascular access in patients with end-stage renal disease (ESRD) plays a crucial role in determining both the quality of life and survival rates, as it directly affects dialysis effectiveness. It connects the patient to the dialysis machine and significantly impacts the dialysis dose by influencing blood flow efficiency. To meet dialysis goals, the required blood flow should be at least 250 mL/minute, and recent guidelines suggest aiming for over 300 mL/minute during a four-hour session [1-13]. Despite advancements in technology, improved care, and collaborative efforts by healthcare providers and patients, complications related to vascular access continue to be a major cause of hospitalizations and mortality among patients with ESRD [1-9]. This ongoing issue also places a considerable financial strain on the healthcare system. As the number of dialysis patients rises, healthcare costs are escalating, underscoring the need for innovations in vascular access that enhance effectiveness, improve survival rates, and prevent complications [2].

Our study aims to evaluate how vascular access impacts the dialysis dose and clinical outcomes, with the ultimate goal of enhancing patients' quality of life while minimizing costs to the healthcare system. We seek to identify new strategies to reduce complications associated with vascular access, which contribute to inadequate dialysis and higher mortality rates, and encourage the integration of these strategies into clinical practice and personalized patient care.

## Materials and methods

This retrospective study was conducted at the Clinic of Nephrology and Dialysis at the University Hospital "St. Marina", Varna, Bulgaria, for five years, from the beginning of January 2017 to the end of December 2021. During this period, the medical records and routine laboratory tests of 87 patients who met the study criteria were reviewed. For the comparative analysis, patients were categorized into two groups based on the type of permanent vascular access: Group 1 consisted of 45 patients with an arteriovenous fistula (AVF), and Group 2 included 42 patients with a permanent tunneled vascular catheter (PC). During the specified period, the above-mentioned indicators were studied, as well as the recorded levels of mortality and hospitalizations, analyzing their relationship with the type of vascular access. The research was approved by a protocol 107/28.10.2021 of the Commission on Ethics of Research at the Medical University “Prof. Dr. Paraskev Stoyanov”, Varna, Bulgaria. This article was previously posted to the same university institutional repository as part of the doctoral dissertation of Diana Nenova submitted in April 2022.

Inclusion criteria required that participants be at least 18 years old, have been on hemodialysis (HD) for more than six months, have minimal residual renal function (defined as diuresis less than 100 ml per day), have permanent vascular access, have received the same starting dose of erythropoiesis-stimulating agents (ESA) (47±3.1 IU/kg), have complete medical documentation.

Exclusion criteria excluded individuals who were under 18 years old, had been on HD for less than six months, had temporary vascular access, had significant differences in the starting dose of ESA (which could affect the results), or had incomplete medical documentation.

As a part of the study, the medical records of all 103 patients in the clinic who had been on dialysis for more than six months were examined, of which 96 met the study criteria. After applying the inclusion and exclusion criteria, all 87 patients who met the criteria were included in the final analysis. Nine patients, with an average age of 57.3 ± 2.6 years, were excluded: five had temporary vascular access, and four had significant differences in their initial ESA doses, which could seriously affect the results.

The dialysis procedures were performed using Fresenius Medical Care 4008 and 5008 series equipment (Fresenius Medical Care AG & Co, Germany) and were standardized to minimize variability. Low-flow polysulfone dialyzers were used: "Etropal" (Etropal JSC, Etropole, Bulgaria), "Diadema" (Etropal JSC), and "Ashahi" (Asahi Kasei Medical Co., Ltd., Tokyo, Japan), with a surface area of 1.8-2.1 m², tailored to each patient’s body surface area. The dialysis regimen followed a conventional schedule, with an average weekly dose of 12 ± 0.3 hours, using bicarbonate dialysate at a dialysate flow rate (Qd) of 500 mL/minute and a blood flow rate (Qb) of 280 ± 52 mL/minute.

To assess dialysis adequacy, the study evaluated the following indices: urea reduction ratio (URR), spKt/V, and nutritional parameters such as normalized protein catabolic rate (nPCR) and serum albumin levels. Hemoglobin levels and the average weekly dose of ESA administered were also recorded, alongside morbidity and mortality rates during the study period. All patients were allocated into the two groups with an identical initial ESA dose of 47 ± 3.1 IU/kg to ensure comparability.

All laboratory tests used in this study were performed routinely according to the guidelines set by the National Health Insurance Fund of the Republic of Bulgaria. Standardized procedures were followed for collecting blood samples using the stop-pump technique, both before and/or after hemodialysis (HD), to minimize the effects of recirculation and urea rebound.

Hemoglobin concentration (g/L) was measured via a complete blood count using the colorimetric method with sodium lauryl sulfate, on a 6-diff hematological analyzer (Sysmex XN1000, Siemens, Germany). The reference range (RR) for hemoglobin was 120-180 g/L, with a target range of 110-120 g/L for patients with ESRD. Urea concentration (mmol/L) was determined using a coupled enzyme reaction with glutamate dehydrogenase (GLDH) through a UV kinetic method on the ADVIA Chemistry 1800 system (Siemens, Germany), with a reference range of 3.2-8.2 mmol/L. Creatinine concentration (mmol/L) was measured using the Jaffe-kinetic method on the same system, with a reference range of 44-115 mmol/L. Serum albumin concentration (g/L) was assessed using a colorimetric method with the selective dye bromocresol green (BCG), also on the ADVIA Chemistry 1800 system, with a reference range of 32-48 g/L.

To eliminate the influence of ultrafiltration (UF) on the results, blood samples for hemoglobin and serum albumin were always taken before the start of the dialysis procedure. Blood samples for urea and creatinine were collected both before and after dialysis to assess dialysis adequacy and nutritional status indicators, including single-pool Kt/V index (spKt/V), URR, and nPCR. The latter were calculated based on the formulas presented in Table 1.

**Table 1:** Dialysis adequacy and nutritional status indices - formulas. spKt/V, single-pool Kt/V; URR%, urea reduction  ratio; nPCR, normalized protein catabolic rate; *t*, dialysis time in hours (h); UF, the volume of ultrafiltration in liters (L); *W*, post-dialysis weight of the patient in kilograms (kg); ln, natural logarithm; Co, pre-dialysis urea nitrogen; *C*, post-dialysis urea nitrogen; Cn, pre-dialysis urea nitrogen from the next HD; *R* = *C*/Co; ID, inter-dialysis time in hours (h)

Indicator	Formula
spKt/V	spKt/V =​ ​-ln(*R *- 0.008 x *t*) + [4-3.5 x *R*] x 0.55 *UF*/*W*
URR%	URR% = 100 x (1 – *C*/Co)
nPCR	nPCR = 0.22 + 0.36 x (Cn - *C*) x 24/*ID*

The data analysis was performed using IBM SPSS Statistics for Windows, Version 20.0 (Released 2011; IBM Corp., Armonk, NY), running on Windows 10 (Microsoft Corporation, Redmond, WA). Descriptive statistics were employed to determine the average levels and variations in quantitative variables, as well as the absolute and relative frequencies in qualitative variables. Parametric methods, such as the Student’s t-test, were used for hypothesis testing where appropriate, while non-parametric methods, such as the Chi-square test, were applied for other comparisons. To assess the likelihood of an outcome in the exposed group compared to the unexposed group, relative risk (RR) analysis was conducted. A *P*-value of less than 0.05 was considered statistically significant.

## Results

The average age of the study population was 59.8 ± 5.1 years. No statistically significant differences were found in either dialysis dose or type of vascular access across different age groups (*P *= 0.103). Among the participants, 33 (38%) were women and 54 (62%) were men. No significant association was observed between gender and the type of permanent vascular access used (χ² = 2.356, *P *= 0.561). Regarding concomitant diseases that could influence the results, no statistically significant differences were observed between the two groups (*P *> 0.05). The prevalence of diabetes mellitus was 21.3% in Group 1 and 22.06% in Group 2 (χ^2 ^= 2.05, *P *= 0.359). Peripheral vascular disease was present in 2.6% of patients in Group 1 and 2.81% in Group 2 (*χ*^2 ^= 2.043, *P *= 0.238). Similarly, the proportion of patients with heart failure was comparable: 31.2% in Group 1 versus 30.2% in Group 2 (χ² = 2.68, *P* = 0.224).

Table 2 presents the results of the variation analysis and Student's t-test for the indicators of adequacy, nutritional status, serum hemoglobin, and applied average weekly erythropoietin (ESA) dose for the period under review.

**Table 2:** Results of the variation analysis and Student's t-test over a five-year period. Group 1: studied patients with arteriovenous fistula; Group 2: studied patients with a permanent tunneled vascular catheter. *n*, number of studied patients; URR%, urea reduction  ratio; spKt/V, single-pool Kt/V - dialysis adequacy index; nPCR, normalized protein catabolic rate; Alb (g/L), serum albumin; Hgb (g/L), hemoglobin concentration; ESA, erythropoiesis-stimulating agent; UI/week, international units per week

Indicator	Group 1 (*n* = 45)	Group 2 (*n* = 42)	*t*-test	*P*-value
URR%	74.8 ± 6.1	68.9 ± 5.3	6.267	<0.0001
spKt/V	1.92 ± 0.6	1.4 ± 0.3	8.632	<0.0001
nPCR	1.19 ± 0.1	1.18 ± 0.09	1.234	0.896
Alb (g/L)	38.24 ± 0.58	37.52 ± 0.61	1.564	0.607
Hgb (g/L)	114.06 ± 10.08	102.03 ± 10.11	7.21	<0.0001
ESA (UI/week)	6138 ± 1246	9826 ± 3036	7.54	<0.0001

Group 1 demonstrated significantly better outcomes. Inadequate dialysis, defined as URR < 65% and spKt/V < 1.2, was observed in only 3.1% of patients, predominantly in Group 2, while just 0.8% of Group 1 patients were affected. In Group 2, the leading causes of inadequate dialysis were catheter dysfunction and catheter-associated infections. In contrast, anastomotic recirculation was the primary cause in Group 1.

The results of the Student's t-test for independent variables revealed a statistically significant difference in serum hemoglobin levels and ESA requirements between the two groups (*P *< 0.05). However, no significant difference was found in serum albumin levels, with the mean value for the observed period being 38.24 g/L in Group 1 and 37.52 g/L in Group 2 (*t *= 1.564, *P *= 0.607). Similarly, no significant difference was observed in the nPCR, with mean values of 1.19 in Group 1 and 1.18 in Group 2 (*t *= 1.234, *P *= 0.896). Despite the randomization of patients to an identical starting dose of fast-acting ESA (47 IU/kg), hemoglobin levels in Group 1 were significantly higher at the end of the study, with a notable reduction in ESA dose compared to Group 2 (*P *< 0.05).

**Figure 1:**
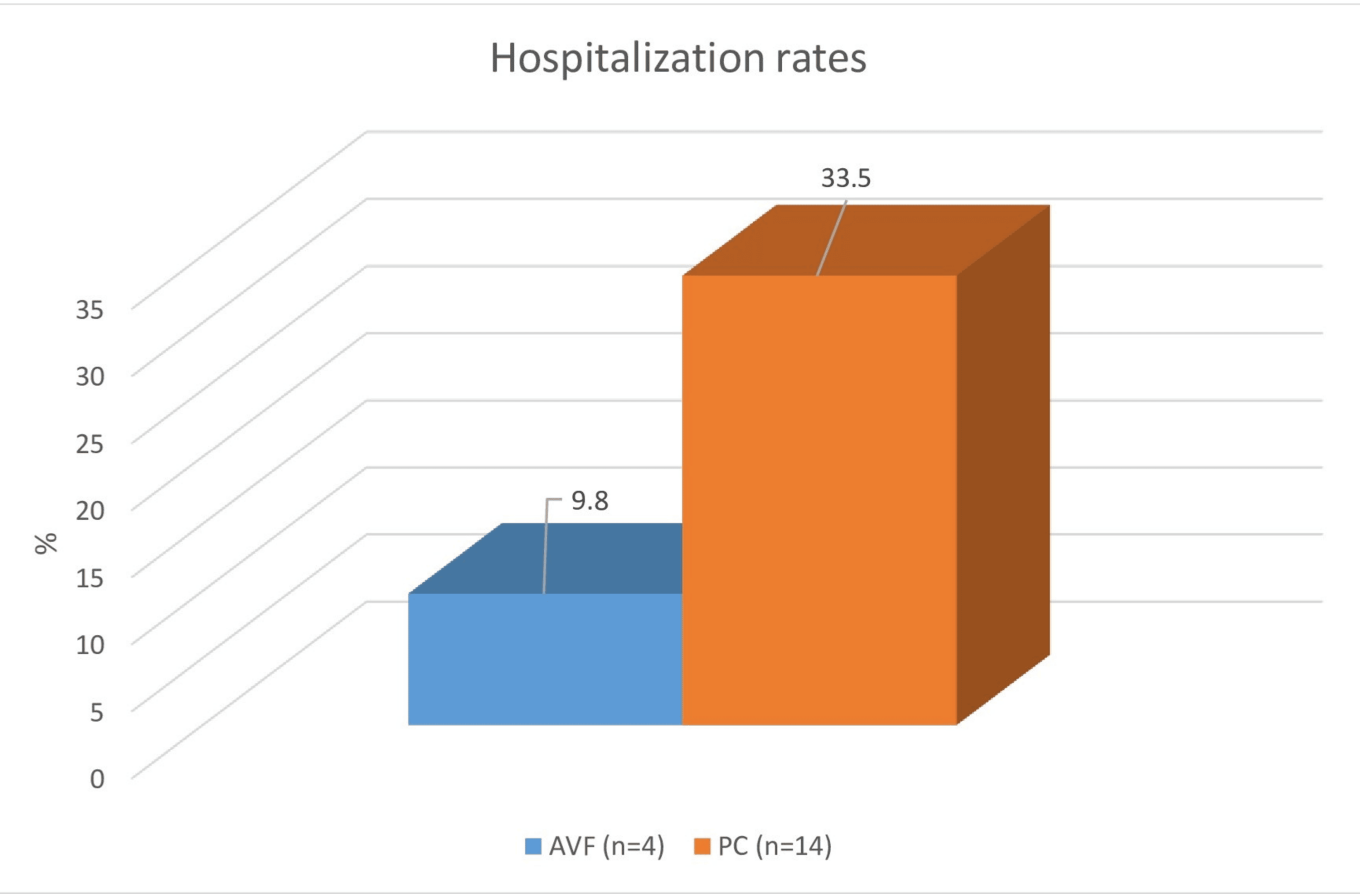
Influence of permanent vascular access on hospitalization rates over a five-year period. Blue column: AVF (arteriovenous fistula); orange column: PC (permanent tunneled vascular catheter); n: number of studied patients

The results also indicated a lower incidence of hospitalizations and mortality among patients with elective vascular access via arteriovenous fistula (AVF). In Group 1, the most common causes of hospitalization were thrombosis of the vascular access and cardiovascular events, occurring in 9.8% of patients. In contrast, in Group 2, the primary causes of hospitalization were catheter-associated infections, followed by cardiovascular and cerebrovascular incidents, with a significantly higher frequency of 33.5% (χ² = 12.867, *P* = 0.002; Figure 1).

**Figure 2:**
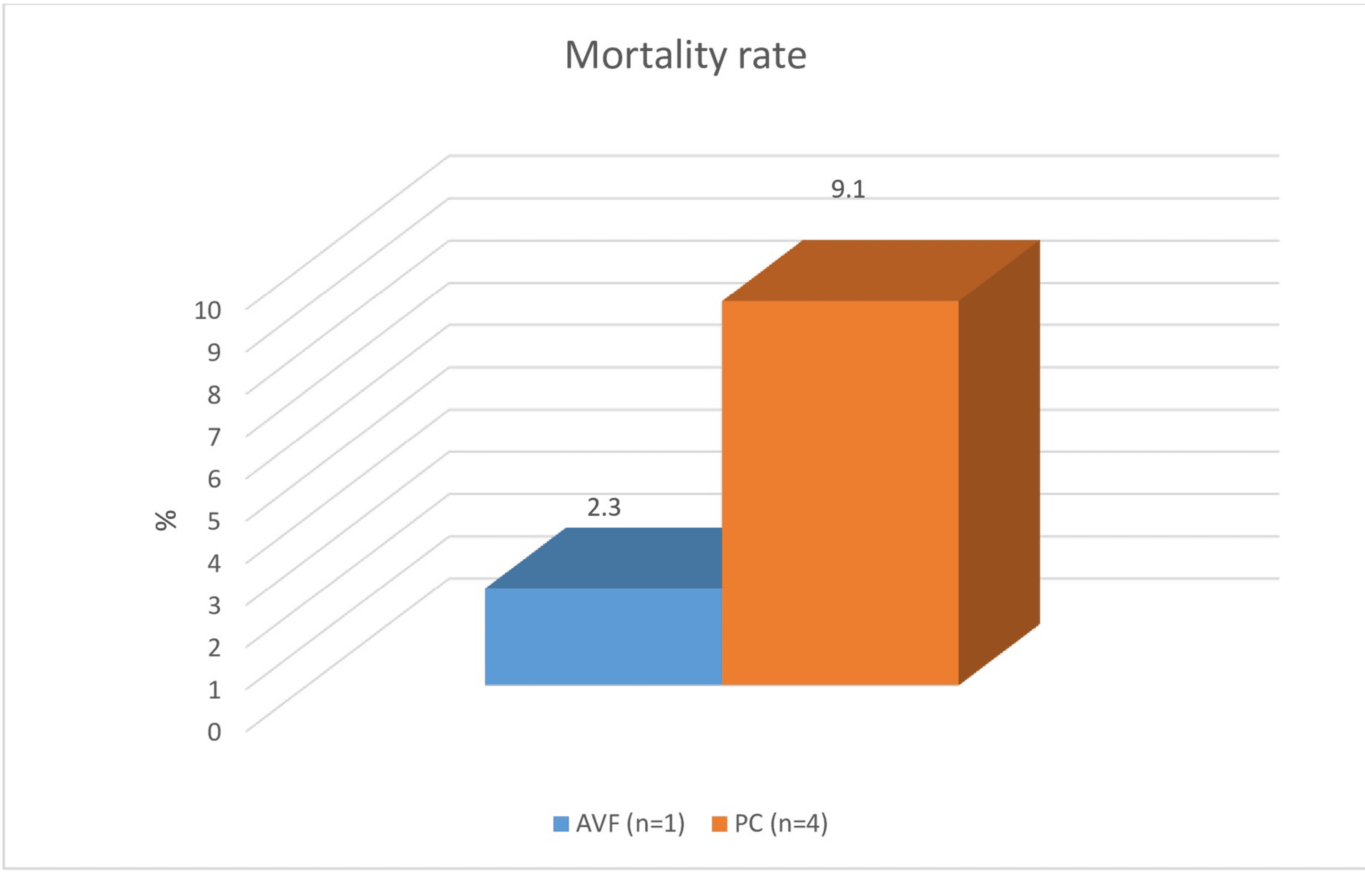
Influence of permanent vascular access on mortality rates over a five-year period. Blue column: AVF (arteriovenous fistula); orange column: PC (permanent tunneled vascular catheter); n, number of studied patients

The overall mortality rate in the study group was 6.3%, with a significantly higher mortality of 9.1% in Group 2 (χ² = 19.24, *P *= 0.001), primarily due to complications from catheter-associated sepsis. In comparison, the mortality rate in Group 1 was 2.3% (Figure 2).

Further analysis revealed that the relative risk of death in Group 2 was 4.09 times higher than in Group 1 (RR = 4.09; 95% confidence interval [CI] 0.4989-36.8151, *P *< 0.001). The most common cause of death in Group 1 was cardiovascular events, which remained the leading cause of death in the dialysis population.

**Figure 3:**
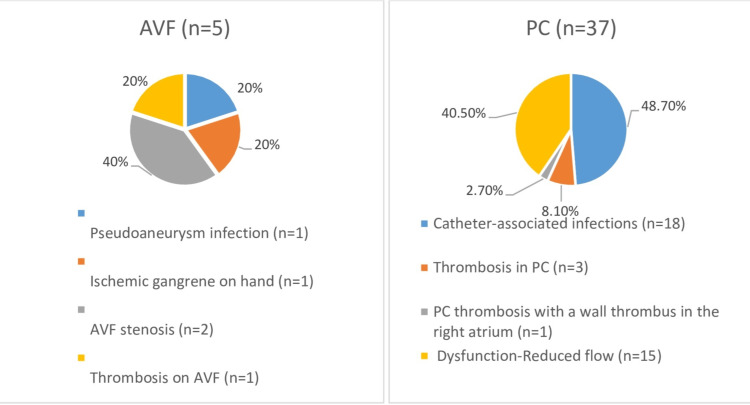
Complications in patients using AVF and PC over a five-year period. AVF, arteriovenous fistula; PC, permanent tunneled vascular catheter; n, number of studied patients

Figure 3 illustrates the most common complications observed in both groups over the five-year follow-up period.

## Discussion

Properly functioning and efficient vascular access is crucial for delivering an adequate dialysis dose. Unfortunately, vascular access remains a significant challenge in hemodialysis, often referred to as the *Achilles' heel* of the procedure. This is due to the high incidence of complications, particularly in patients with permanent catheters, which continue to be one of the leading causes of morbidity and mortality in patients with ESRD. Our findings support this observation, confirming that vascular access-related complications, such as thrombosis, stenosis, access infections, and reduced blood flow requiring temporary catheter replacement, contribute to inadequate dialysis dosing and are a major factor influencing patient outcomes, including an increased risk of cardiovascular events.

Various studies have evaluated the impact of vascular access types on the dialysis dose delivered [2-10]. Theoretically, all things being equal, eliminating other factors influencing dialysis dose such as dialyzer surface area and permeability, active and frequency of dialysis procedures, dialysis flow rate, and with the same value of blood flow effects, e.g., 300 mL/minute, there should be no difference in the delivered dialysis dose. However, the reported results are quite contradictory. A two-year study by Canaud et al. on 42 hemodialysis patients reported that dialysis adequacy (measured by spKt/V) was slightly lower in PC compared to AVF, and to compensate for this, longer dialysis sessions were suggested for patients with PC [2,6]. Furthermore, Canaud et al. compared dialysis adequacy and mean blood flow in patients, and the results obtained reported the advantage of AVF over other types of vascular access and confirmed that overall dialysis adequacy and mean blood flow in AVF were significantly higher compared to PC and vascular prostheses as permanent vascular access. Similar results were reported by Ethier et al. in a multicenter study in several countries, showing that dialysis adequacy in patients with AVF was higher compared to patients with PC [2,7]. In addition to the report that after AVF, the preferred vascular access is a vascular prosthesis. According to Karkar et al., in a study of 358 hemodialysis patients, an increase in AVF usage was associated with a reduced incidence of infection and access thrombosis, increased mean blood flow, decreased mean spKt/V, lower hemoglobin levels alongside reduced erythropoietin doses, improved serum albumin levels, and a significant reduction in hospitalization rates [2,8].

Our analysis demonstrated the significant superiority of AVFs in several key areas, including dialysis dose, survival rates, complication incidence, and overall quality of life. Specifically, patients with AVF showed significantly higher dialysis adequacy, as measured by spKt/V and urea reduction ratio (URR), along with higher serum hemoglobin levels and lower erythropoietin (ESA) requirements. Moreover, the AVF group experienced fewer complications and lower mortality rates, consistent with the findings of Karkar et al., Canaud et al., Ethier et al., Momeni et al., and Vachharajani et al. [2,6-10].

However, our results contrast with studies by Mutavelic et al. and Chiu et al., who reported no significant differences in dialysis dose or other clinical indicators between patients with AVF and those with permanent catheters [11,12]. These discrepancies may be attributed to differences in patient populations, study designs, or healthcare settings, highlighting the need for further research to fully understand the factors influencing vascular access outcomes in patients with ESRD [2].

Overall, our study reaffirms the importance of AVF as the preferred choice for vascular access in hemodialysis, given its superior performance in terms of dialysis adequacy, reduced complications, and improved patient survival.

The reported incidence of inadequate dialysis in our study was relatively low, but it was primarily observed in patients with permanent catheters (PC). The main reason for this was catheter dysfunction, particularly the inability to achieve a blood flow rate of 250 mL/minute or higher [1,2,13,14]. This issue is often related to improper positioning of the catheter during placement, but over time, it can also be caused by stenosis, thrombosis, incorrect heparinization, or damage such as twisting or breaking of the catheter in the subcutaneous tunnel [2,15]. Catheter-associated infections also contributed to the reduced dialysis dose observed in the PC group. Infections often necessitate the replacement of the catheter with temporary vascular access, which typically offers lower blood flow and consequently reduces dialysis efficiency [2].

In contrast, the most common complications in the AVF group were stenosis and thrombosis, which are typical of vascular anastomoses. However, the frequency of these complications is much lower compared to those associated with PC, as confirmed by our findings and those of other authors [2,9,10,16-21]. While AVF-related complications, such as pseudoaneurysms, are less frequent, they can still occur. Pseudoaneurysms are a rare complication that may lead to dysfunction, rupture, or infection. Other complications in patients with AVF include skin necrosis due to repeated puncture of the same sites (which can be complicated by life-threatening bleeding), ischemia of the hand that may progress to gangrene, and hyperdynamic syndrome, which can lead to heart failure due to increased blood flow through the anastomosis [9,14,15,22,23]. These complications, though rare, may necessitate the placement of a temporary central venous catheter (CVC), which again leads to reduced blood flow, lower dialysis doses, and worsened clinical outcomes [2].

The significantly higher incidence of complications in the PC group is well-documented in the literature and is directly linked to an increased rate of hospitalizations and mortality in this group. These outcomes are primarily due to access-related events, but also reflect the broader impact of compromised dialysis adequacy. Inadequate dialysis, in turn, worsens clinical outcomes, potentiating issues such as malnutrition, chronic inflammation, and immune dysfunction [2,11,14,20,24]. These factors collectively contribute to the poor prognosis observed in patients with PC, reinforcing the importance of optimal vascular access in improving patient survival and quality of life [2].

Some studies dispute a link between the type of vascular access and the control of anemic syndrome, a topic that remains widely debated due to inconsistent outcomes [11,12]. According to Mutavelić et al. and Chiu et al., despite significantly higher mortality and hospitalization rates among patients with indwelling catheters, no significant differences were observed in blood flow rates, serum hemoglobin levels, or the use of medications to manage anemia [11,12]. This seemingly contradictory finding may be attributed to several limitations: a small sample size, lack of randomization to a standardized starting dose of erythropoiesis-stimulating agents (ESAs), pre-existing correction of iron deficiency at baseline, and a relatively short follow-up period of only two years. These factors limit the comparability of results between the two patient groups due to the absence of uniform study conditions. However, our analysis demonstrates not only improved hemoglobin levels but also a lower required erythropoietin (ESA) dose in patients with AVFs, with a statistically significant relationship. The better control of anemia in Group 1 (patients with AVF) is primarily attributed to the higher dialysis dose received. In contrast, the higher incidence of infections in patients with PC, along with chronic inflammation and blood loss from frequent clotting in catheter dysfunction, contributes to persistently lower hemoglobin levels in Group 2. This, in turn, necessitates the use of significantly higher doses of ESA to achieve target hemoglobin levels.

In addition to the well-established benefits of AVF in terms of dialysis adequacy, attention must be drawn to the significantly higher survival rates associated with AVF use [2,20,25-28]. Our analysis reveals a significant difference in mortality rates between the two groups. This difference is mainly due to the combined effect of the lower dialysis dose in patients with PC and the high frequency of catheter-associated sepsis, which is the leading cause of death in this group. In contrast, the primary cause of death in the AVF group was cardiovascular complications. This strongly suggests that the type of vascular access used is linked to mortality, not only indirectly through the dialysis dose received but also directly through the complications associated with vascular access. The causes of mortality in the PC group are mainly related to access infections, whereas in the AVF group, mortality causes are more consistent with those seen in the general dialysis population. Thus, patients in the PC group are at a significantly higher risk of death related to access, with the risk being four times greater than in patients with AVF [2].

The growing trend of mass placement of PCs is concerning, as evidenced by the 2019 United States Renal Data System (USRDS) report [29]. In our clinic, only 30% of patients are dialyzed through AVF, which is a troubling statistic. The benefits of higher dialysis doses in patients with AVF, better anemia control, and a lower incidence of complications and hospitalizations extend beyond physical health. They also positively impact the subjective emotional component of quality of life, including self-esteem, mental health, and overall well-being. In contrast, frequent flow issues, infections, the need for catheter repositioning, and reliance on temporary catheters all significantly impair the quality of life for patients with PC. These challenges can lead to feelings of frustration, anxiety, and depression, further deteriorating their condition and contributing to a vicious cycle where patients lose motivation to maintain their health and well-being [2].

Limitations

This study has several limitations, including the exclusion of pediatric patients, a lack of patients presenting with arteriovenous graft as a type of vascular access, a focus on five years, a small representative sample, and the study only included patients from a single clinic.

## Conclusions

While PCs may still be necessary in certain situations, our findings reinforce the superior outcomes associated with AVF use in terms of dialysis adequacy, survival, anemia control, and overall quality of life. It is essential to prioritize AVF creation whenever possible to reduce complications and improve the long-term prognosis for patients with ESRD.

Despite the recent KDOQI 2019 vascular access guidelines, which emphasize an individualized approach and consider the patient's personal preferences, we maintain that AVF should remain the preferred choice in all suitable cases. To achieve this, it is essential to promote AVF as the primary vascular access option while educating patients about its benefits and potential risks. Additionally, a multidisciplinary approach should be employed, one that incorporates updated algorithms for the creation, maintenance, and management of AVF, as well as early detection and timely treatment of complications.

We also advocate for the development of a standardized algorithm for nursing care that unifies the processes of vascular access management. Such standardization would enhance the preservation of AVFs and help prevent infections. As is well known, there are significant variations in how vascular access is maintained, both between different dialysis centers and among individual healthcare professionals. Therefore, implementing standardized procedures would not only improve the consistency and quality of medical care but also lead to significantly better clinical outcomes for patients. This approach has the potential to maximize the benefits of AVF, reduce complications, and improve patient survival and quality of life in the long term.
